# A survey of statistics in three UK general practice journal

**DOI:** 10.1186/1471-2288-4-28

**Published:** 2004-12-13

**Authors:** Alan S Rigby, Gillian K Armstrong, Michael J Campbell, Nick Summerton

**Affiliations:** 1 Academic Cardiology, University of Hull, Kingston-upon-Hull, UK; 2 NAPP Pharmaceuticals Research Limited, Cambridge, UK; 3 Medical Statistics Unit, University of Sheffield, Sheffield, UK; 4 Division of Primary Care & Psychological Medicine, University of Hull, Kingston-upon-Hull, UK

## Abstract

**Background:**

Many medical specialities have reviewed the statistical content of their journals. To our knowledge this has not been done in general practice. Given the main role of a general practitioner as a diagnostician we thought it would be of interest to see whether the statistical methods reported reflect the diagnostic process.

**Methods:**

Hand search of three UK journals of general practice namely the *British Medical Journal (general practice section)*, *British Journal of General Practice *and *Family Practice *over a one-year period (1 January to 31 December 2000).

**Results:**

A wide variety of statistical techniques were used. The most common methods included t-tests and Chi-squared tests. There were few articles reporting likelihood ratios and other useful diagnostic methods. There was evidence that the journals with the more thorough statistical review process reported a more complex and wider variety of statistical techniques.

**Conclusions:**

The *BMJ *had a wider range and greater diversity of statistical methods than the other two journals. However, in all three journals there was a dearth of papers reflecting the diagnostic process. Across all three journals there were relatively few papers describing randomised controlled trials thus recognising the difficulty of implementing this design in general practice.

## Background

"Diagnosis is the keystone of good medical practice"[1]

General practitioners (GPs) are primarily diagnosticians [2] yet it appears that diagnosis remains their Achilles heel[3]. The problem has its origins in a misunderstanding of the differences of the five Ps (patients, pathologies, presentations, prevalences and predictive values) in hospital practice compared to primary care[4]. Decisions made by GPs are different from those made by hospital clinicians. The precise diagnostic labels may be less important than deciding on an appropriate course of action. Hence, diagnoses are often framed in terms of binary decisions; treatment versus non-treatment, disease versus non-disease, referral versus non-referral, and serious versus non-serious for example[4].

From a statistical viewpoint the binary decision making process has a lot of appeal. For example, the use of the naïve Bayes' discriminant function (and from it the derivation of likelihood ratios) is appropriate. Proponents of Bayes' argue for its simplicity and ease of interpretation[5,6]. In contrast, opponents argue that data are not used efficiently if they are simply ploughed through the "black box" of Bayes'[7,8]. Whatever the rights and wrongs of Bayes' as a technique it is time for GPs to become more familiar with statistical methods aimed at diagnosis. In relation to haematuria (blood in the urine) and the diagnosis of urological malignancy two of the authors of this paper (NS and ASR) have used Bayesian techniques in order to seek to refine diagnostic discrimination by general practitioners [9]. The results from this work have been incorporated successfully into local primary care oriented referral guidance.

Many medical journals, both generalist[10,11] and specialist [12-18], have been reviewed for their statistical content. Articles have been published in the fields of radiology, [12-14] otolaryngology, [15,16] rehabilitation medicine[17] and ophthalmology[18] to name but a few. However, general practice is under researched in this area[19]. The aim of this paper is to review three leading UK journals in general practice and to see what statistical methods are being used. It is not our intention to see if the methods are being used correctly but to look at the range of techniques reported. The outcome of this research should give pointers to the future education of GPs who wish to undertake research.

## Methods

Three statisticians (MJC, ASR and GKA) (two of them holding Chartered status of the Royal Statistical Society) including one Professor, one Senior Lecturer and one Lecturer each reviewed one leading UK journal in general practice. The fourth author (NS) is a Primary Care Physician. The journals chosen were the *British Medical Journal (BMJ) (general practice section)*, *British Journal of General Practice (BJGP) *and *Family Practice*. These three journals were chosen because they reflected the main primary care journals in the UK. The journals were hand searched for original research articles over a one-year period (1 January to 31 December 2000). Articles were classified for both their statistical content and methods of design according to criteria laid down elsewhere[10,20]. Tables 1 and 2 list the classification criteria used for both study design and statistical methods. Letters were excluded on the grounds that they are typically responses to previously published material rather than original contributions in themselves. We are aware, of course, that not all primary care research is published in these three journals alone and we comment on this later.

**Table 1:** Classification of design methods (after Wang and Zhang, 1988) [19]

Design method

Case report
Cross-sectional survey
Retrospective study
Prospective study
Clinical trial
basic science study

**Table 2:** Classification of statistical methods (after Emerson and Colditz, 1983) [10]

Category	Brief description
No statistical methods or descriptive statistics	No statistical content, or descriptive statistics only (e.g., percentages, means Standard deviations, standard errors, histograms
Contingency tables	Chi-square tests, Fisher's test, McNemar's test
Multiway tables	Mantel-Haenszel procedure, log-linear models
Epidemiological studies	Relative risk, odds ratio, log odds, measures of association, sensitivity, specificity
t-tests	One-sample, matched pair, and two sample t- tests
Pearson correlation	Classic product-moment correlation
Simple linear regression	Least-squares regression with one predictor and one response variable
Multiple regression	Includes polynomial regression and stepwise regression
Analysis of variance	Analysis of variance, analysis of covariance, and F-tests
Multiple comparisons	Procedures for handling multiple inferences on same data sets (e.g., Bonferroni techniques, Scheffe's contrasts, Duncan's multiple range procedures, Newmann-Keuls procedure)
Non-parametric tests	Sign test, Wilcoxon signed ranks test, Mann- Whitney test, Spearman's rho, Kendall's tau, test for trend
Life table	Actuarial life table, Kaplan-Meier estimates of survival
Regression for survival	Includes Cox regression and logistic regression
Other survival analysis	Breslow's Kruskal Wallis, log rank, Cox model for comparing survival
Adjustment & standardisation	Pertains to incidence rates and prevalence rates
Sensitivity analysis	Examines sensitivity of outcome to small changes in assumptions
Power	Loosely defined, includes use of the size of detectable (or useful) difference in determining sample size
Transformation	Use of data transformation (e.g., logs) often in regression
Cost-benefit analysis	The process of combining estimates of cost and health outcomes to compare policy alternatives
Other	Anything not fitting the above headings includes cluster analysis, discriminant analysis, and some mathematical modelling

The main study was preceded by a pilot phase in which a random sample of 10 articles was classified both by statistical content and study design by the three statisticians. Where there were differences of opinion, consensus was reached by discussion. We met once to discuss our classification system, and to iron out differences of opinion. One problem lay in how we actually classified study design. For example, one of use used the phrase 'cross-sectional survey' while another used the phrase 'questionnaire survey' when both meant the same in terms of study design. Another problem was that we missed some of the statistical techniques (where there were many) and this required much more careful reading of the articles when we carried out the main survey. We did not carry out a formal reliability study of the pilot phase but instead relied on our experiences both as statisticians, and as journal reviewers. Similarly we chose not to carry out a formal reliability analysis in the main study.

## Results

The total number of articles reviewed over a one year period was as follows: *BMJ *(general practice section) (n = 79), *BJGP *(n = 145) and *Family Practice *(n = 81).

### Study design

**Figure 1:**
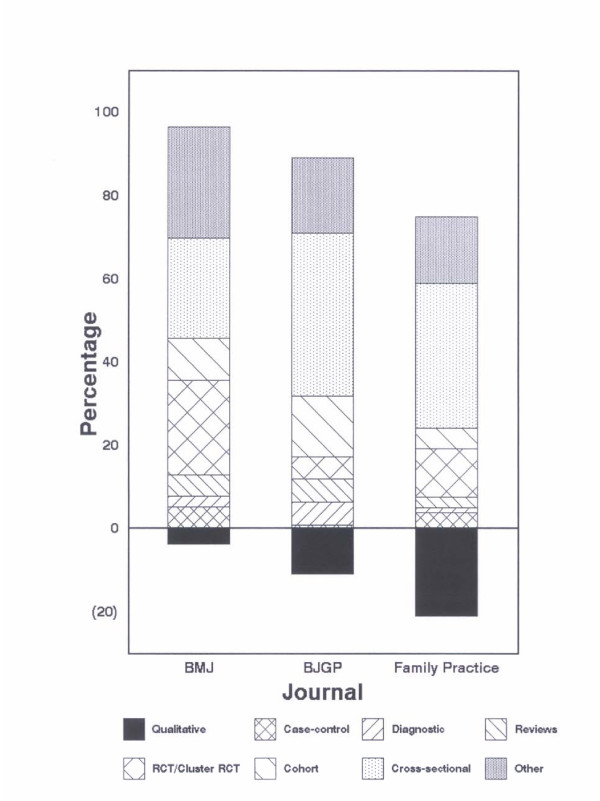
Proportion of papers ranked by a qualitative design

The most common design was that of a cross-sectional survey being found in 24.1%, 39.3% and 35.1% of articles in the *BMJ*, *BJGP and Family Practice *respectively (Table 3). Although we classified articles by the term 'cross-sectional survey' this was not necessarily the choice term adopted by the journal. Sometimes the phrase 'questionnaire survey' was used and we assumed this was data collected cross-sectionally. We found a similar difference in nomenclature for our phrase 'cohort study' in which the phrase 'prospective survey' was also found. The highest proportion of qualitative studies was in *Family Practice *(21.0% compared to an average of 11.8%). Qualitative studies included those encompassing terms such as 'focus groups' and 'semi-structured interviews' for example. Figure 1 shows the proportion of papers ranked by a qualitative design. For all three journals, diagnostic studies were infrequently used. Examples of these include those based on screening (e.g., the usefulness of N-terminal brain natriuretic peptide level for screening of patients with heart failure), and calculating the sensitivity and specificity of diagnostic tests (e.g., *Helicobacter pylori *for the detection of peptic ulcer). Examples of more unusual study designs include those based on video recordings, literature reviews and quasi-experimental designs.

**Table 3:** Design methods

	BMJ		BJGP		Family Practice		Overall	
	
Designs	n	(%)	n	(%)	n	(%)	n	(%)
Cross-sectional survey	19	(24.1)	57	(39.3)	31	(34.8)	107	(35.1)
Qualitative study	3	(3.8)	16	(11.0)	17	(21.0)	36	(11.8)
Cohort study	8	(10.1)	21	(14.5)	4	(4.9)	33	(10.8)
RCT	14	(17.7)	7	(4.8)	8	(9.9)	29	(9.5)
Reviews	4	(5.1)	8	(5.5)	2	(2.5)	14	(4.6)
Reliability/diagnostic	2	(2.5)	8	(5.5)	1	(1.2)	11	(3.6)
Case-control study	4	(5.1)	1	(0.7)	3	(3.7)	8	(2.6)
Cluster RCT	4	(5.1)	1	(0.7)	2	(2.3)	7	(2.3)
Other	21	(26.6)	26	(17.9)	13	(16.0)	60	(19.7)
Total articles	79		145		81		305	

Note

RCT = randomised controlled trial.

### Statistical methods

The range of statistical methods reported can be seen in Table 4. The number of methods exceeds the number of articles as some reported more than one technique. There are differences between the journals. The *BMJ *shows a greater range and breadth of articles than *Family Practice*. More sophisticated techniques are reported more often in the *BMJ *than either of the other two journals. In the *BMJ*, the two most common statistical methods used were logistic regression (n = 14, 17.7%) and the Chi-squared test (n = 13, 16.5%). The two least common were the Mantel-Haenszel statistic (n = 1, 1.3%) and Cronbach's alpha (n = 1, 1.3%). Relatively new innovations such as random effects models were seen in both the *BMJ *and the *BJGP*. The least sophisticated statistical methods appeared in *Family Practice*. Methods based on likelihood ratios were seldom found in either the *BMJ *or *BJGP *and not at all in *Family Practice*. Nonparametric tests were often unspecified but where they were included Mann-Whitney U test, Spearman's correlation coefficient and the Wilcoxon matched-pairs signed ranks test. Multiple comparisons included Bonferonni techniques and Scheffe's contrasts. Survival analysis included Kaplan-Meier curves and Cox regression.

**Table 4:** Statistical methods

	BMJ		BJGP		Family Practice		Overall	
	
Methods	n	(%)	n	(%)	n	(%)	n	(%)
No statistics or simple summaries	23	(29.1)	47	(32.4)	33	(40.7)	103	(33.8)
Chi-squared tests	13	(16.5)	40	(27.6)	19	(23.5)	72	(23.6)
t-tests	7	(8.9)	22	(15.2)	17	(21.0)	46	(15.1)
Logistic regression	14	(17.7)	19	(13.1)	11	(13.6)	44	(14.4)
Nonparametric	11	(13.9)	24	(16.6)	4	(4.9)	39	(12.8)
Odds ratios/relative risks	11	(13.9)	13	(9.0)	14	(17.3)	38	(12.5)
Regression	9	(11.4)	10	(6.9)	11	(13.6)	30	(9.8)
Sample size/power	6	(7.6)	17	(11.7)	3	(3.7)	26	(8.5)
Summaries with CIs	9	(11.4)	3	(2.1)	6	(7.4)	18	(5.9)
Kappa	2	(2.5)	9	(6.2)	4	(4.9)	15	(4.9)
Sensitivity/specificity	4	(5.1)	10	(6.9)	1	(1.2)	15	(4.9)
Pearson correlation	2	(2.5)	6	(4.1)	6	(7.4)	14	(4.6)
Multiple comparisons	2	(2.5)	4	(2.8)	4	(4.9)	10	(3.3)
ANOVA	5	(6.3)	4	(2.8)			9	(3.0)
Mantel-Haenszel	1	(1.3)	5	(3.4)	2	(2.5)	8	(2.6)
Random effects models	4	(5.1)	4	(2.8)			8	(2.6)
Cronbach's alpha	1	(1.3)	5	(3.4)	1	(1.2)	7	(2.3)
Fisher's exact test			7	(4.8)			7	(2.3)
Likelihood ratio	3	(3.8)	3	(2.1)			6	(2.0)
Survival analysis	6	(7.6)					6	(2.0)
Other	4	(5.1)	37	(25.2)	10	(12.3)	51	(16.7)
Total articles	79		145		81		305	

Notes

CIs = confidence intervals.

ANOVA = analysis of variance.

One-third of all articles reported no statistics or simple summaries (for example, mean, median, percentage, standard deviation, interquartile range). No journal article with a qualitative design had any statistical content.

A large number of articles reported other statistical methods, in particular the *BJGP*. This was due to a wide range of statistical methods being reported only once. Examples include time series, multilevel modelling and factor analysis. In others, we could not decipher which statistical techniques had been used.

Table 5 shows the rank order of the statistical methods by each journal. Differences between the journals can be seen more clearly.

**Table 5:** Ranking of statistical techniques

	BMJ	BJGP	Family Practice
	
Methods	Rank	Rank	Rank
Chi-squared tests	2	1	1
t-tests	7	3	2
Logistic regression	1	4	4.5
Nonparametric	3.5	2	9
Odds ratios/relative risks	3.5	6	3
Regression	5.5	7.5	4.5
Sample size/power	8.5	5	11
Summaries with CIs	5.5	17	6.5
Kappa	15	9	9
Sensitivity/specificity	11.5	7.5	13.5
Pearson correlation	15	11	6.5
Multiple comparisons	15	15	9
ANOVA	10	15	
Mantel-Haenszel	17.5	12.5	12
Random effects models	11.5	15	
Cronbach's alpha	17.5	12.5	13.5
Fisher's exact test		10	
Likelihood ratio	13	17	
Survival analysis	8.5		

Notes

Excluding other methods and no statistics/simple summaries.

CI = confidence interval.

ANOVA = analysis of variance.

## Discussion

Two-thirds of all journal articles relied on some type of statistical analysis beyond descriptive statistics (Table 4). The Chi-squared test and t-tests were commonly used in the *BJGP *and *Family Practice*. Papers in the *BMJ *and the *BJGP *used more sophisticated statistical methods than *Family Practice *(Table 4). While both the *BMJ *and the *BJGP *used sophisticated methods, the *BMJ *used them more often. Why might this be so? The sophistication of methods used is influenced by three factors. First, issuing instructions to authors of a statistical nature. This requires a bank of statisticians available for review to which the *BMJ *has access. Second, general articles on statistical aspects of writing papers. Third, tutorial type articles explaining specific techniques. The *BMJ *continues to take a lead in the latter two areas and indeed published statistical guidelines for contributions to medical journals over 20 years ago[21]. Despite the lack of sophistication in *Family Practice*, there has been a trend of using more advanced statistics elsewhere,[14,15,17,20] and this has been linked to the increasing availability of computer packages[14]. The *BJGP *is currently struggling to find statistical reviewers (personal communication by Editor to ASR). It is perhaps too easy for us to lay blame at the Editors door for this lack of sophistication. Statisticians are relatively rare, and review, for the most part, is unpaid.

Although these three journals publish a large proportion of the research in general practice within the UK, they by no means represent 100% of it. To look at this further we examined the year 2000 and undertook a MEDLINE search using the key indexing phrase 'General Practice'. We found over 800 articles in a diversity of journals. Articles were published in the fields of rheumatology, medical ethics, obstetrics, public health, clinical pharmacology, clinical neurology and telemedicine to name but a few.

We chose to look at the year 2000. Would our results be different had we selected a different year? The published literature suggests otherwise. In a 20 year old study, Emerson and Colditz[10] found t-tests (44%) and Chi-squared tests (27%) were the most common statistical methods reported although now Chi-squared tests are more common than t-tests (Table 5). Given the emphasis on statistical computing today we might have expected less reliance on these two methods. What lies behind this lack of progress? Altman and Goodman[22] looked at the speed of the transfer of technology of new statistical methods into the medical literature. They concluded that many methodological innovations of the 1980s had still not made their way into the medical literature of the 1990s suggesting a typical lag-time of 4–6 years. Lag-time is likely to be related to quality statistical review and this may be longer in journals with less impact. It is also worth reporting that since we carried out this survey (year 2000) there will have been a modest increase in the use of newer, more sophisticated statistical techniques.

Now let us turn to study design. The gold standard research design is considered to be the randomised controlled trial (RCT). It has been acknowledged that carrying out RCTs in general practice are difficult[23,24]. In our survey we found few RCTs (Table 3). There are particular problems of recruitment with respect to primary care. Many issues have been discussed. For example, most practices have no formal contractual arrangement to participate in research and may be unwilling to participate unless there is immediate benefit to their patients. It is known that motivating practices for long-term follow-up studies particularly is not easy[25]. Practices may feel uncomfortable about randomising their patients[26] but, delegation of this duty to another may lead to a breakdown of the special doctor/patient relationship. There are statistical and sample size concerns also. Randomisation by practice (so-called cluster randomisation) leads to larger sample sizes being required[27,28].

What are the issues here? Are they really that different from secondary care? A recent publication posed the question 'What do residents really need to know about statistics?'[29]. The authors surveyed six journals and catalogued them for their statistical and methodological content. The most popular statistical tests across the whole range of journals were the Chi-squared test followed by the t-test. The authors concluded that with knowledge of each of these two tests clinicians should be able to interpret up to 70% of the medical literature.

## Conclusions

For all three journals there was a dearth of articles reflecting the diagnostic process. Why is this? It has already said that diagnosis is the Achilles Heel of GPs[3]. If it is not to remain this way we must start to educate doctors. The question is how. The latest "Tomorrow's Doctors"[30] states that students must have "Adequate knowledge of the sciences on which medicine is based and a good understanding of the scientific methods including principles of measuring biological functions, the evaluation of scientifically established facts and the analysis of data". Clearly, there is a role for teaching statistics in the education of doctors who wish to undertake research. The much greater prevalence of methods concerning binary data (Chi-squared test, logistic regression, odds ratios/relative risks) over methods concerned with continuous data should be reflected in our (statistical) teaching. Initial training in means, medians and modes should be replaced by relative risk, absolute risk and numbers needed to treat.

## Competing interests

The author(s) declare that they have no competing interests.

## Authors' contributions

Three authors (ASR, GKA and MJC) carried out the literature review while all four authors contributed to the writing.

## Pre-publication history

The pre-publication history for this paper can be accessed here:



## References

[B1] Anonymous (1987). Diagnosis: logic and pseudo-logic. The Lancet.

[B2] Morrell DC (1993). Diagnosis in General Practice. Art or Science?.

[B3] Howie JGR (1972). Diagnosis – the Achilles Heel?. Journal of the Royal College of General Practitioners.

[B4] Summerton N (1999). Diagnosing Cancer in Primary Care.

[B5] Hilden J (1984). Statistical diagnosis based on conditional independence does not require it. Computational Methods in Biology and Medicine.

[B6] Crichton NJ, Fryer JC, Spicer CC (1987). Some points on the use of 'Independent Bayes' to diagnose acute abdominal pain. Statistics in Medicine.

[B7] Feinstein AR (1977). The haze of Bayes, the aerial palaces of decision making and the computerised Ouija board. Clinical Pharmacology and Therapeutics.

[B8] Morton BA, Teather D, du Boulay GH (1984). Statistical modelling and diagnostic aids. Medical Decision Making.

[B9] Summerton N, Mann S, Rigby AS, Ashley J, Palmer S, Hetherington JW (2002). Patients with new onset haematuria in relation to urological cancer attending an 'open access' clinic: assessing the discriminant value of items of clinical information in relation to urological malignancies. British Journal of General Practice.

[B10] Emerson JD, Colditz GA (1983). Use of statistical analysis in the *The New England Journal of Medicine*. New England Journal of Medicine.

[B11] Ripoll RM, Terren CA, Vilalta JS (1996). The current use of statistics in biomedical investigation: A comparison of general medicine journals. Medicina Clinica.

[B12] Elster AD (1994). Use of statistical analysis in the AJR and Radiology- Frequency, methods and subspeciality differences. American Journal of Roentgenology.

[B13] Goldin J, Zhu W, Sayre JW (1996). A review of statistical analysis used in papers published in Clinical Radiology and British Journal of Radiology. Clinical Radiology.

[B14] Golder W (1999). Statistical analyses in German radiological periodicals: The last decade's development. Rofo-Fortschritte auf dem gebiet der rontgenstahlen und der bildgebdenden verfahren.

[B15] Rosenfeld RM, Rockette HE (1991). Biostatistics in otolaryngology journals. Archives of Otolaryngology, head and neck surgery.

[B16] Bhattacaryya N (1999). Peer review: Studying the major otolaryngology journals. Laryngoscope.

[B17] Schwartz SJ, Sturr M, Goldberg G (1996). Statistical methods in rehabilitation literature: A survey of recent publications. Archives of Physical Medicine and Rehabilitation.

[B18] Juzych MS, Shin DH, Seyedsadr M, Siegner SW, Juzych LA (1992). Statistical techniques in ophthalmic journals. Archives of Ophthalmology.

[B19] Thomas T, Fahey T, Somerset M (1998). The content and methodology of research papers published in three United Kingdom primary care journals. British Journal of General Practice.

[B20] Wang Q, Zhang BH (1998). Research design and statistical methods in Chinese medical journals. Journal of the American Medical Association.

[B21] Altman DG, Gore SM, Gardner MJ, Pocock SJ (1983). Statistical guidelines for contributions to medical journals. British Medical Journal.

[B22] Altman DG, Goodman SN (1994). Transfer technology from statistical journals to the biomedical literature – Past trends and future predictions. Journal of the American Medical Association.

[B23] Pringle M, Churchill R (1995). Randomised controlled trials in general practice. Gold standard or fool's gold ?. British Medical Journal.

[B24] Sheikh S, Smeeth L, Ascroft R (2002). Randomised controlled trials in general practice: scope and application. British Journal of General Practice.

[B25] Tognoni G, Alii C, Avanzini F, Bettelli G, Colombo F, Corso R (1991). Randomised controlled trials in general practice: lessons from failure. British Medical Journal.

[B26] King M, Broster G, Lloyd M, Horder J (1994). Controlled trials in the evaluation of counselling in general practice. British Journal of General Practice.

[B27] Donner A, Brown KS, Brasher P (1990). A methodological review of non-therapeutic intervention trials employing cluster randomisation, 1979–1989. International Journal of Epidemiology.

[B28] Campbell MJ (2000). Cluster randomised controlled trials in general (family) practice. Statistical Methods in Medical Research.

[B29] Reed III, Salen P, Bagher P (2003). Methodological and statistical techniques: what do residents really need to know about statistics?. Journal of Medical Systems.

[B30] General Medical Council (2002). Tomorrow's doctors Recommedations on undergraduate medical education.

